# A Message from the Editor-in-Chief for *Pharmacy*—A Journal of Pharmacy Education and Practice

**DOI:** 10.3390/pharmacy9020073

**Published:** 2021-03-31

**Authors:** Jon C. Schommer

**Affiliations:** College of Pharmacy–Twin Cities, University of Minnesota, 308 Harvard Street, SE, Room 7-155 WDH, Minneapolis, MN 55455, USA; schom010@umn.edu; Tel.: +612-626-9915; Fax: +612-625-9931

Dear Reader of *Pharmacy*,

It is an honor to begin serving a two-year term as Editor-in-Chief for *Pharmacy* (ISSN 2226-4787), a journal of pharmacy education and practice. It is an international scientific open access journal, published quarterly in an online format by MDPI (Multidisciplinary Digital Publishing Institute). It brings together research on the practice of pharmacy with research on the education and development of the pharmacy workforce and the impact of health policy and government on pharmacy healthcare delivery [1]. *Pharmacy* was established in 2013 and supervised by founding Editor-in-Chief, Prof. Dr. Keith A. Wilson from Aston University, UK until his retirement in 2021. With profound gratitude, we thank Professor Wilson for his vision and leadership. He cast a vision with an external focus on the “other” instead of the “us” to establish the value of the journal to readers and their needs. That external focus helped meet the needs of society as well as the profession of pharmacy. Through his leadership, he developed a healthy organization and structure with processes and procedures designed to support authors and reviewers. More recently, the journal intentionally and successfully reached out globally to help integrate more and more scholars in this endeavor worldwide. Submission and publication metrics continue to rise and article processing times continue to shorten. A highly professional staff and editorial team are in place for supporting the publishing process.

So, what comes next? An endeavor at this stage of development is ready to integrate and welcome new participants. Expansion into more countries, cultures, systems of care, and patient populations can help promote health equity so that each individual has a fair and just opportunity to be as healthy as possible. This is a laudable goal worldwide, in my opinion. Furthermore, expanding our research in pharmaceutical product development, information systems, and pharmaceutical care practice is needed. These are well established in pharmacy and building upon these foundations will be fruitful. In addition, research regarding Coordinated Healthcare through Integration of the Medication Experience (CHIME) is a newer phenomenon and is crucial. CHIME reflects a person’s regular interaction with medications as the most frequently and consistently occurring health care event in one’s life. Additionally, it interfaces with almost all other aspects of a person’s health care. As health-care systems move away from fragmented approaches and closer to a team-based, patient-centered care approach, there is a need to unify and coordinate individuals’ health care even as these individuals enter and exit various components of the health-care system and as they shift between their preferred identity as a person and their sometimes-necessary identity as a patient. An individual’s subjective experience of taking medications in daily life can be a unifying and coordinating concept to bridge this dichotomy and the pharmacist’s role is central in this domain. The development of this area is timely and imperative. 

Pharmacy education and practice are increasingly coordinated in their roles for taking medications from bench to bedside, to best practice, to best outcome, and best value. This involves natural, clinical and social sciences, including the humanities. Pharmacy covers the full spectrum of translational science where basic science research is translated into new methods of diagnosis, treatment and prevention of diseases in humans. This work is translated to patient care and practice guidelines through clinical trials and evidence-based research. Finally, patient care guidelines developed under controlled conditions are translated into individualized patient care and benefit to society through population health. Translational research across these domains is needed to help optimize the role of pharmaceuticals and the pharmacy practitioner in new and old systems of health care. 

Another area of expansion for our journal can be the continual pursuit of developing the future of pharmacy. Van Antwerp proposed that “exponential change is accelerating disruption across the health care value chain and transforming the future of pharmacy” [2]. He predicts rapid disruptions in areas such as gene therapy, microbiomics, digital therapeutics, central-fill delivery hubs, ingestible robotics, home health diagnostics, automated artificial intelligence algorithms, smart home technology, and social determinants of health. Transformation in pharmacy practice and education will need to be bold and dynamic to meet these changes and the opportunities they unfurl. This journal can serve as a venue for discourse and the reporting of findings in emerging domains. 

In summary, I believe that *Pharmacy* can continue to expand in the areas of (1) health equity, (2) coordinated care, (3) translational sciences, and (4) disruptive change that creates transformative opportunities. As we work together, several values that I learned from my experiences in graduate education [3] can serve us well:

- Mentoring one another to become better scholars, leaders, and educators.
- The conduct and application of theory-driven research.
- Enhanced publishing experiences to ensure the development of strong scholars and leaders.
- Transformations in pharmacy practice and policy.
- Diversity of people, cultures, health systems, and methods of inquiry.
- Engagement at local, national, and international levels.
- Collaboration.
- Continued development of individuals and professional advancement.

I look forward serving in this role and to what the future holds.

Jon Schommer, RPh, PhDProfessor, University of Minnesota, USA
